# ‘I knew I had to drive my own bus’: perspectives of patients and healthcare professionals on improving information provision to rare cancer survivors

**DOI:** 10.1007/s11764-025-01896-0

**Published:** 2025-09-19

**Authors:** Tamsin Farrugia, Saskia F. A. Duijts, Christine Cockburn, Laura Hemming, Carlene Wilson, Evelien Spelten

**Affiliations:** 1 Violet Vines Marshman Centre for Rural Health Research, Rural Health School, La Trobe University, Bendigo, VIC Australia; 2 Department of Research and Development, Netherlands Comprehensive Cancer Organisation (Integraal Kankercentrum Nederland, IKNL), Utrecht, The Netherlands; 3 Department of Medical Psychology, Amsterdam University Medical Center, Location Vrije Universiteit, Amsterdam, The Netherlands; 4 Cancer Center Amsterdam, Cancer Treatment and Quality of Life, Amsterdam, The Netherlands; 5 Rare Cancers Australia, Bowral, NSW Australia; 6 Melbourne School of Population and Global Health, The University of Melbourne, Melbourne, Australia

**Keywords:** Rare cancer, Information needs, Information provision, Supportive care, Oncology

## Abstract

**Purpose:**

People diagnosed with a rare cancer report a high need for information. Research on rare cancer survivors’ preferences for, and challenges in, accessing information is scarce. The aims of this study were to explore the following: (1) rare cancer patients’ preferences for information content and delivery mode and (2) the experiences of healthcare professionals in delivering information to this patient group.

**Methods:**

Interviews with rare cancer survivors and healthcare professionals were conducted between October 2023 and June 2024. Semi-structured interview guides were developed for both survivors and professionals. Interviews were recorded, transcribed verbatim and analysed using thematic analysis.

**Results:**

A total of 32 interviews (20 survivors and 12 healthcare professionals) were undertaken. Four themes were generated: (1) ‘Questions need to be addressed regardless of information availability’, (2) ‘Information seeking is a fundamental part of the rare cancer experience’ , (3) ‘Healthcare professionals are responsible for information sharing’ and (4) ‘Tailoring information to patient preferences is a balancing act’.

**Conclusion:**

This study highlights the importance of providing tailored, relevant information to rare cancer survivors in a face-to-face format, despite there being a paucity of information available on many rare cancers. Survivors highlighted the importance of their role in sourcing information and advocating for themselves, whereas health professionals identified their responsibility for providing tailored information to their patients.

**Implications for Cancer Survivors:**

The findings suggest a need to improve how information is structured and delivered to rare cancer survivors to ensure their unique needs and questions are adequately addressed.

## Introduction

Rare cancers are defined as those with an incidence fewer than 6 diagnoses per 100,000 people per year [1]. Individually, rare cancers are a large group of heterogeneous diseases but, collectively, they are a great burden on the healthcare system and are responsible for 15% of all cancer deaths [1]. Due to the rarity of their disease and the associated poor understanding of their illness in the health and wider communities, rare cancer patients face many challenges, including worse medical outcomes (e.g., lower survival rates [2]) and significant psychosocial challenges (e.g., higher level of uncertainty and lower quality of life [3]), compared to those diagnosed with a common cancer. Limited clinical expertise about many rare cancers and a paucity of evidence to guide decision making are significant challenges.

The provision of evidence-based information to a cancer patient has been shown to improve emotional well-being [4, 5]. Particularly for those diagnosed with a rare disease, information provides a sense of empowerment, providing people with the necessary tools to advocate for their care [6]. However, with limited clinical expertise and awareness of rare cancers among general healthcare providers, rare cancer patients have reported low satisfaction with the information provided [7], with some being forced to search for information themselves [8, 9]. Ultimately, there is limited research exploring access to information by rare cancer patients, despite information needs being described as the greatest unmet supportive care need for these patients [10].

By contrast, for common cancer patients, unmet information needs have been well documented and researched [11]. For example, it is understood that common cancer patients prefer information provided by their primary physician [12, 13]. The information is generally easy to obtain and most indicate satisfaction with the information received about their diagnosis [14].

Perceptions of the importance of timely information provision and the difficulties in sourcing and sharing it have also been explored among healthcare professionals treating people with common cancers [15]. The perceptions of those treating people with a rare cancer are less well documented. Vandeborne et al. [16] reported that knowledge and awareness of rare disease amongst physicians was suboptimal and education on rare disease management and information sources was needed. A full nuance of rare cancer patients’ attitudes to information provision is required to improve health professionals’ understanding of how to best deliver relevant information to their patients.

To address these gaps in research, our study aimed to understand the current environment in which information about rare cancer is sought and obtained by interviewing rare cancer survivors and healthcare professionals. Specifically, we aimed to explore survivors’ preferences for information type, how it should be received and from whom. In addition, the challenges healthcare providers face in meeting the information needs of people with a rare cancer were also explored. 

## Methods

### Design

Data comprised qualitative, semi-structured interviews with rare cancer survivors (*n* = 20) and healthcare professionals (*n* = 12) involved in the management of people in Australia diagnosed with a rare cancer. This study was approved by the Human Ethics Committee of La Trobe University (HEC23234).

### Participant recruitment and procedure

Participants were selected using maximum variation sampling to ensure perspectives were captured for patients varying by age, gender, employment, location (rural versus urban) and rare cancer type. Patients were eligible to participate if they were (1) adults (≥ 18 years) currently living with a rare cancer, who received their diagnosis during adulthood (≤ 5 years ago), and (2) able to communicate fluently in English. Healthcare professionals were eligible to participate if they were (1) a professional specialising in the management and care of patients diagnosed with a rare cancer and (2) able to communicate fluently in English.

Participants were recruited through Rare Cancers Australia’s (RCA) internal database of rare cancer patients and healthcare professionals. In the first instance, the RCA’s Patient Support Team contacted potential participants to gauge their interest and availability to participate. Participants who expressed their interest in participating were emailed an invitation letter and a Participant Information and Consent Form (PICF) in which the aims and procedures of the study were explained. Patients were also emailed a link to a short online questionnaire to capture demographic information. Information about healthcare professionals was collected during the interviews. Upon return of the completed documents, interviews were scheduled based on the participant’s availability. Recruitment of participants ceased when no new themes or patterns were identified. All invitations and responses were managed by one researcher (TF).

### Data collection

Semi-structured interviews were conducted by the researcher (TF) via Zoom video conferencing. At the commencement of each interview, the participant’s consent was reconfirmed, and the aims and study procedure were reiterated. All participants were able to withdraw from the study at any point in the interview and up until 4 weeks following their interview date. All participant information was kept confidential.

Two different interview guides (Appendix 1 and 2) were followed to ensure relevant information was gathered for each sample. The interview guide for people living with a rare cancer was co-designed with members from RCA’s Patient Advisory Board, a consumer-led team of patients and people affected by a rare cancer. Co-design of the patient interview guide ensured that the interview questions were meaningful and appropriate. The interview guide for healthcare professionals was developed by all researchers (TF, SD, CC, CW, ES), four of whom (SD, CC, CW, ES) have extensive experience in conducting qualitative research. All interviews with rare cancer survivors were followed up via phone call by one Specialist Cancer Navigator from RCA to provide additional support to the participant and answer any concerns or address any distress felt by the participant during or after the interview.

### Data analysis

All interviews were audio recorded and transcribed verbatim for analysis. Data were analysed using thematic analysis and NVivo software (version R1/2020) to facilitate the coding and analysis process. The researcher (TF) independently coded each interview transcript, producing preliminary themes from both patients’ and healthcare professionals’ interviews. Preliminary themes were discussed comprehensively between all authors (TF, SD, CC, LH, CW, ES) until refinement and consensus about the main themes were reached. Sociodemographic characteristics of the patient group were retrieved from the online questionnaire and reported in descriptive statistics (Table 1).

**Table 1:** Patient participant characteristics (*n* = 19)

Characteristic	*n*	%
Age		
< 45 years	6	31.6
45–64 years	9	47.4
≥ 65 years	4	21.0
Gender		
Female	13	68.4
Male	6	31.6
Indigenous status		
Do not identify	18	94.7
Identify as Aboriginal and/or Torres Strait Islander	1	5.3
Place of residence		
Metropolitan	17	89.5
Regional	2	10.5
Level of education		
Secondary school	4	21.0
Apprenticeship/TAFE course	4	21.0
University	11	57.9
Cancer diagnosis		
Adrenocortical carcinoma	1	5.3
Blood cancer	2	10.5
GIST	2	10.5
NUT carcinoma	1	5.3
Rare female genital cancer	2	10.5
Sarcoma	5	25.3
Thyroid cancer	2	10.5
Thymic gland	4	21.1

## Results

### Participant characteristics

Thirty-two interviews were conducted between October 2023 and June 2024. Patient interviews (*n* = 20) lasted between 23 min and 1 h and 31 min (mean 51 min) and included six male (aged between 26 and 71 years, mean 48) and 14 female (aged between 32 and 72 years, mean 51) participants. A total of eight rare tumour types were identified, with sarcoma being the most frequent diagnosis (*n* = 5). Most patients resided in metropolitan areas (*n* = 17) and only one participant identified as Aboriginal and/or Torres Strait Islander. Demographic data from one participant were not provided. Further information about patients’ characteristics can be found in Table 1.

Interviews with healthcare professionals (*n* = 12) lasted between 19 and 52 min (mean 34 min) and involved five male and seven female participants. Disciplines included cancer navigator/coordinator (*n* = 3), medical oncologist (*n* = 2), neurosurgeon (*n* = 2), nurse (*n* = 2), radiation oncologist (*n* = 1), social worker (*n* = 1) and surgical oncologist (*n* = 1). All professionals were located in metropolitan areas, with Sydney, NSW (*n* = 8) being the most frequently reported area of work.

### Key findings

Following data analysis, four main themes were constructed: (1) Questions need to be addressed regardless of information availability, (2) information seeking is a fundamental part of the rare cancer experience, (3) healthcare professionals are responsible for information sharing and (4) tailoring information to patient preferences is a balancing act.

#### Questions need to be addressed regardless of information availability

Many survivors described how the rarity of their disease meant that limited information was available to them before and after diagnosis. Despite this, participants understood the importance of receiving relevant and personalised information in order to improve their understanding of their situation. Some participants described their frustration in not receiving answers to their questions about their condition, noting that this resulted in confusion and uncertainty.

“When the crisis occurs, such as a cancer diagnosis, one of the most important things that people need is good, accurate, and timely information.” – P03, male, aged 71 years

“I was told, ‘write down all your questions’ … so I did. I really thought about it, I wrote down all my list of questions and I asked each team this list of questions … I was basically told you know that, no they weren’t going to answer them, they could answer them but they chose not to, or they didn’t know the answer to them, or they wouldn’t give me hypotheticals … So that was also very frustrating.” – P06, male, aged 53 years

One consequence of navigating an information-poor environment was a degree of confusion among survivors about who to contact and when, with some interviewees describing their inability to discern a serious event from an expected outcome. In these situations, the sense of isolation and lack of control were heightened, with uncertainty about the future a common consequence of not knowing who to contact:

“I just don’t understand, and I don’t know who to talk to. This all an information-based thing. Who do I go to? Who do I ask?” – P09, female, aged 72 years

“When to call? Should I call? Is this a triple 0 [emergency] call? Or is this a nurse call, or is it, just suck it up, Princess, and deal with it?” – P12, male, aged 49 years

There was acknowledgement of this confusion from some healthcare professionals, who recognised the challenge for the patient in knowing who to speak to about their care. Health care interviewees indicated it was important for them to reassure the patient about their ongoing commitment and involvement in the patient’s cancer management, while also ensuring the patient received optimal care.

“It can be confusing sometimes. And so that’s why I say, ‘Yeah, I’m still part of your team. But the person who’s calling the shots is the radiation specialist… and although it might be easier for you to ask me, cause you’re sitting here in front of me today, that’s not the right way for you to get the best coordination of your care’.” – HP12, male, Neurosurgeon

In the absence of information, the uncertainty associated with a rare cancer diagnosis was exacerbated, with some survivors discussing their fear about the future and current state of ambiguity. In these situations, survivors reflected on their situation as one requiring tolerance and patience.

“Just not knowing what lays ahead. You don’t have that much information on the cancer. I feel like I’m just a sitting duck waiting to find out what the next move is … moving forward is only about one step ahead every time. There’s never anything beyond that … unfortunately it’s a battle, a war that you just gotta take things as it comes…” – P17, male, aged 35 years

“I think it’s the fear … when someone’s told they got a rare cancer, you’ve got that fear of, well there’s nothing they can do about it because it’s rare they don’t know what to do. … They don’t know how to treat it. And you gotta be used as a guinea pig. I think that’s the worst thing thinking that you know they’re going to be trialling all these different things on you because they don’t know what to give you.” – P18, female, age unknown

#### Information seeking is a fundamental part of the rare cancer experience

People diagnosed with a rare cancer emphasised the importance of their own endeavours in finding information about their diagnosis, highlighting how the rarity of their disease magnified the challenge. Often, this personal search for information reflected the poor availability or provision of specific information through health providers:

“So it was, just thrown back on me then to do my own research to get the information which I was looking for.” – P03, male, aged 71 years

“With rare cancers you don’t have that gold standard protocol... I knew I had to drive my own bus.” – P10, female, aged 55 years

For many participants, information seeking was seen as empowering and a way of capturing control of the disease and, therefore, life. Several survivors discussed the importance of seeking information for their own understanding and sense of control:

“But having the information for myself was very empowering. And it allowed me to also talk about things that are greater level and understanding…” – P10, female, aged 55 years

“…the most important thing is just being able to arm yourself with information about your disease, your potential diagnosis and … what your journey looks like moving forward… knowledge is power … even if it is confronting I still want to know…” – P15, male, 52 years

Additionally, survivors reflected on the benefits of information seeking. Beyond personal knowledge, information enabled some participants to help others by sharing their findings, raising awareness through contributing to the body of information, and creating a sense of hope for others going through a similar experience:

“If I could help in any way that would be amazing. I’ve done something.” – P09, female, aged 72 years

“Anything that I can do to voice, how I’m going through with this journey and my positivity, if I can share that with other people and give them hope and support in any way. That’s my day done… that’s my good deed for the day.” – P17, male, aged 35 years

Conversely, some survivors described information-seeking as tiring and difficult when their diagnosis was poorly understood. They expressed a sense of disappointment and frustration with the need to take responsibility for finding information themselves:

“I just found out so much information but had there been a checklist it would have helped me … At times I felt quite exhausted … I was really, really, just so tired.” – P02, female, aged 68 years

“I didn’t have the inclination or the energy for any more research … And by the time you do have the energy again, you’re kind of through it.” – P14, female, aged 47 years

Most survivors reported they actively used the internet to find information about their cancer, with the greatest benefit of online searching creating the opportunity to connect with others going through a similar experience. Sourcing information from others with a shared experience relieved feelings of uncertainty and diminished perceived isolation. Consequently, peer connection and support from someone else diagnosed with a rare cancer were viewed as particularly important for reducing feelings of isolation and ‘aloneness’ as well as providing the opportunity to share information and broaden knowledge about the specific rare cancer. However, survivors expressed that the timing of peer connection was crucial; the individual had to be emotionally ready to connect with others:

“I’m very grateful and thankful that I’ve found a group on Facebook of the cancer … And that’s where I’ve gotten my information about it the most.” – P17, male, aged 35 years

“I think people who understand how hard it is it’s been really good. But I had to be ready emotionally for that … It can be bit triggering … I reached out when I felt like I needed to.” – P11, female, aged 34 years

Both groups of participants reported a general sense of apprehension when sourcing information online, acknowledging the risks of exposure to unreliable material. Their hesitation in trusting online information stemmed from the broad volume of conflicting information available and uncertainty about their diagnosis and difficulty in finding information on the specific rare tumour type.

“I ended up, you know, you Google, and you research... And sometimes that information isn’t accurate … Google’s very literal. You can’t just take it at face value … so that information is not always helpful, but that’s all I had.” – P14, female, aged 47 years

Healthcare professionals also discussed the difficulty associated with their patients relying on information from the internet, acknowledging the challenge in identifying reliable and relevant information. Professionals were particularly concerned that their patients might be overwhelmed by information that was personally irrelevant or misleading.

“The Internet is unregulated.... there’s a whole lot of stuff out there that is not credible and can be misleading and distressing.” – HP03, female, Oncology Social Worker

“Most people get the information from the Internet these days.... And Internet, you know, it’s a two-edge sword... you have to know whether the information you’re reading is high quality or not, that’s not easy to do... even if it is high quality, is it relevant to you ‘cause that’s not easy... it’s not written for you as an individual it’s written for a group of people with a condition. And your own circumstances may be different.” – HP05, male, Medical Oncologist

#### Healthcare professionals are responsible for information sharing

Many patients indicated they relied on their clinician as the primary source of information, particularly seeking guidance from their treating oncologist. Healthcare professionals recognised their responsibility for providing relevant and reliable information to their patients, emphasising the importance of not encouraging patients to seek information on their own.

“The clinician who’s giving them the diagnosis should have most of it [responsibility], because how they frame giving the diagnosis is important because that will influence how the patient receives it.” – HP02, female, Clinical Nurse Consultant

Although healthcare professionals were seen as having the sole responsibility for information provision, this did not always extend to responsibility for treatment decisions. Some survivors desired ownership over their treatment path, valuing consent and shared decision-making.

“It’s informed consent… I think it is the right thing that the patient makes that decision not just somebody saying, you’ve got a [specific rare tumour], you now have to be on this drug.” – P02, female, aged 68 years

This was also acknowledged by many professionals, who, despite their influence on treatment decisions, respected shared decision-making and patient choice. In some circumstances, professionals reported the challenge of their patients trying alternative (or overseas) treatment and the difficulty of managing this from a medical and emotional perspective. In these situations, professionals conveyed their respect for these decisions but indicated concern and disappointment when the patient ignored local support.

“I think the responsibility for the patient [is] to realise that yeah, the medical system is working with their best interest in mind, and they need to turn up. So the worst thing is when people decide not to turn up or decide to take what I call alternative therapies. I’m very happy that they take complimentary therapies. I say … ‘that’s fine, you know, but don’t try and replace what we’re trying to offer you with something else.’” – HP10, male, Radiation Oncologist

Above all, trust was highly valued by both survivors and healthcare providers. When a person was not offered a clear treatment path, trust and confidence in the healthcare provider were impacted. Some survivors also found themselves in the role of providing, rather than receiving, information, which impacted trust in their clinical team. In these situations, survivors felt overwhelmed with this new responsibility and were determined to ensure their medical team was educated on the rare cancer type.

“I got all these other oncologists but, all kept saying was ‘oh gee! This is rare, this is rare. What do I do? What sort of treatment am I gonna give you?’. And so I lost a lot of confidence in a lot of oncologists because they didn’t know what to do.” – P18, female, age unknown

“I had so many medical students come up and talk to me. “Do you mind if we talk to you? It’s very rare that we have someone like you in” and I said, “learn from me because someone I don’t want anyone else to go through what I’ve just gone through.” – P19, female, aged 71 years

When healthcare professionals acknowledged their limitations and conveyed this appropriately, trust was established. Limited clinical expertise was acknowledged by some professionals, who explained the importance of recognising their limitations and referring to another discipline when a rare cancer, outside their scope of expertise, was presented to them.

“We have to know all about it [a particular rare diagnosis] otherwise we wouldn’t be doing what we do. So I think it’s important that we recognise when our patients have diseases outside of our area that we need other people’s help with. … quite often there are uncertainties that haven’t been appropriately resolved by the right kind of trials yet, but in terms of knowing what data there is, it's our job to know that.” – HP09, male, Surgical Oncologist

Overall, there was great respect among patients for healthcare professionals managing rare cancers. However, there was also a general sense of disappointment when professionals provided only clinical information. Survivors wanted emotional support from providers, reflecting on their need for empathy and validation.

“I needed someone to say ‘Look, it’s normal for you to feel this way. It’s a scary time. It’s this, this and this. And these are strategies to help you, and you will go through these emotions and this is what you can expect and blah blah blah’, That kind of thing I needed from someone.” – P01, female, aged 45 years

Finally, the importance of finding an appropriate clinician to manage their cancer diagnosis was a priority for survivors because this influenced their cancer experience and trajectory. Identifying an appropriate clinician however was a challenge for some survivors. Healthcare professionals indicated recognition of this and identified it as their responsibility to support patients navigate the system.

“I always try and make sure that the patients know who’s running the their treatment coordination at that time. And it will shift over the course of their treatment.” – HP12, male, Neurosurgeon

#### Tailoring information to patient preferences is a balancing act

Participants’ responses in relation to the amount of information desired differed between survivors and healthcare professionals. Many survivors reported wanting to receive as much information as possible, indicating indifference as to whether their healthcare provider agreed with them or trusted them with that information. This was usually driven by a sense of uncertainty and desire to have some control over their diagnosis.

“More information the better because then I can make a sound judgment on what I’ve been presented.” – P12, male, aged 49 years

“It would just be amazing if you could just say, ‘I want everything, I don’t think I don’t care if you think it’s relevant or not or if I can handle it, I just want to do that part myself’.” – P07, male, aged 26 years

However, healthcare professionals reported caution in providing their patients with information, suggesting that being on the receiving end of a life-changing diagnosis could impact an individual’s ability to process information and determine what is important and relevant to them.

“I’ve seen, you know, highly intelligent people live in very highly achieving jobs, who are completely bamboozled about the management of their condition … they don’t have the right background to sort of understand which parts of it are important, what are not. Which things are influential in decision making, what aren’t.” – HP05, male, Medical Oncologist

“I think that’s very difficult for patients to understand the nuances of rare tumours. Again, I think that’s a bit different compared to common cancers.… Because the amount of information out there … it may not be relevant in their particular circumstance.” – HP11, male, Neurosurgeon

Survivors also indicated a desire for information on very specific aspects of their diagnosis, indicating disappointment when this information was not provided by their healthcare provider. There was also some frustration in not being provided the desired information at a particular point in time and in a form acceptable to the individual.

“There’s a lot of information out there it’s not always accessible at the time that you need it, and it’s not always written in a form that is suited to you at the time.” – P20, female, aged 33 years

Ultimately, the way in which information was tailored to patients was critical to ensure their understanding of, and satisfaction with, the information received. Many patients noted the difficulty in interpreting medical information, highlighting how this impacted their capacity to share information with family members. Although some patients noted their health background helped them understand medical language and statistics, they also acknowledged circumstances would have been different for someone with a different education.

“If I wasn’t a clinician who understood health literature, who understood what it was to read research papers and understand what’s a credible source and what’s not, it would have been very overwhelming.” – P11, female, aged 34 years

Healthcare professionals also acknowledged the importance of providing necessary medical information, consistent with the patient’s health literacy. Ascertaining this was a critical precondition to information provision.

“… giving them information appropriate to their information needs and their health literacy. So you don’t want to overwhelm people with stuff that is just going to make them frightened of overwhelmed.” – H03, female, Oncology Social Worker

There was a shared preference for face-to-face communication by patients and professionals. Healthcare professionals indicated the importance of using the first in-person consultation to establish trust. In-person communication was preferred by both groups when difficult or unexpected news was to be shared, with some professionals emphasising the importance of involving a support person in consultations, at this time, in order to mitigate the pressure of information overload on the stressed patient.

“The first patient interview you gotta see them face to face … and take a long time and really develop a deep rapport. Because at the end of the day, it’s all about patient trust, winning patient trust.” – HP10, male, Radiation Oncologist

“… often these patients, as I said, they’re a bit shell shocked, because sometimes they’re given, you know, a worse prognosis, and … they’re a bit lost. So they often don’t hear the message. So, having a support person there or on the phone, I think hopefully strengthens that communication with the patient.” – HP11, male, Neurosurgeon

## Discussion

### Main findings

This study describes the experiences of rare cancer survivors and healthcare professionals managing rare cancers, when seeking or providing information about their cancer and the associated challenges. Both survivors and professionals acknowledged the paucity of information available on many rare cancers, reflecting on the challenges this created. Survivors highlighted the importance of their role in sourcing information and advocating for themselves, while also expressing the need for answers and honesty from their medical team. Professionals acknowledged their responsibility for providing tailored information to their patients, identifying the challenges inherent in balancing essential with desired information.

### Interpretation of findings

Paucity of information provision was an important common thread throughout participants’ experiences, with survivors reflecting on the frustration this generated. Specifically, by not receiving answers to their questions, survivors were left with greater uncertainty about their disease trajectory and often felt displaced in the healthcare system. This is not uncommon for people diagnosed with a rare disease, with previous studies reporting on the challenge in finding disease-specific, high-quality and credible information [17, 18], in which uncertainty about disease progression negatively affects emotional well-being and health-related quality of life [19]. In this study, survivors reflected on their confusion in navigating the healthcare system, particularly with regard to who they should contact in the event of an emergency. Previous research has also shown that the most frequently reported unmet supportive care need for rare cancer patients is in the information and health system domain [10].

Survivors reflected on the need to become an ‘expert’ in their disease because of the scarcity of available literature, a common finding amongst patients with rare diseases [20]. They relied primarily on the internet for information about their diagnosis, with this mirroring findings from previous studies of patients diagnosed with a rare disease [8, 17, 21]. Participants reflected on the utility of the internet for finding information; however, they also acknowledged the need for caution in order to avoid misinformation. This view was mirrored by health professionals. Previous research also suggests cancer patients face difficulties in navigating online information because the information may be complex, written in unfamiliar medical terms, or because the reader feels unsure of how to assess the relevance and reliability of the information [12, 22]. One positive aspect of the online information-seeking behaviour of cancer survivors noted in the study was the opportunity to connect with peers, with survivors observing that relationships made with others sharing a similar experience reduced feelings of isolation. The importance of peer support for cancer patients cannot be underestimated; research has shown it to improve cancer knowledge, psychological empowerment [23] and learning self-management techniques [17]. However, in the present study, and in previous research [24], the desire to connect with peers varied and was determined by an individual’s emotional capacity and readiness.

All participants reflected on the challenge of healthcare professionals providing tailored information to their rare cancer patients. They noted the difference between the information wanted by their patients and that deemed necessary by healthcare professionals. Many survivors indicated wanting comprehensive information, and the ability to decide whether information was relevant to them. Healthcare professionals, however, expressed the potential harm that might result if providers offered too much information to their patients. The optimisation of information provision has been explored in previous studies [25], suggesting that too little, or too much, information can make it difficult for patients to make informed decisions about their care. Survivors also reported a preference for in-person communication. This aligns with previous research with cancer patients that demonstrates the effectiveness of this mode of information in reducing unmet information needs and improving satisfaction with information provided [26].

Many survivors indicated healthcare providers were the preferred source of information. This aligns with previous studies [12, 27] and emphasises the importance of healthcare professionals in meeting the information needs of their patients. Similar to previous studies [28, 29], the findings emphasised the importance of a trusting relationship between the healthcare provider and cancer patient, with survivors desiring empathy and honest information from their provider, even when available information was negative. Simons et al. (2024) reported that rare cancer patients value receiving confirmation of professional expertise from their healthcare provider to establish trust, as well as transparency about available information [29]. Research also suggests that the way in which information is communicated to cancer patients can cause harm, especially when providers show a lack of concern for patients’ needs [25]. Healthcare professionals managing rare cancer patients should therefore be abreast of the current information about rare cancers while maintaining honest communication with their patients, in order to ensure trust and minimise uncertainty.

### Strengths and limitations

To our knowledge, this is the first qualitative study exploring preferences for, and challenges in, information provision for people diagnosed with a rare cancer, from both the patient and health professional perspectives. A few other studies published have reported views from participants diagnosed with one type of rare cancer [8, 30]; our study includes a range of rare cancers. This provides a unique perspective, highlighting how rarity rather than specific cancer type is an important consideration when examining the patients’ experience.

A limitation of this research is the geographical homogeneity of the sample. Although we aimed to achieve maximum variation sampling, participants were predominantly located along the east coast of Australia in metropolitan areas, therefore providing a perspective that might differ from those living in more rural areas of Australia. All professionals interviewed were located in, and offered care to, patients in metropolitan cities. Although professionals indicated they consulted with patients across Australia, the fact that they worked in large cities may limit their capacity to empathise with the challenges faced by those with a rare cancer diagnosis living outside large cities. Furthermore, the demographic characteristics of the survivor cohort may have influenced the findings, with only one participant identifying as Indigenous.

Finally, participants were recruited through one patient support organisation (Rare Cancers Australia). Although partnering with Rare Cancers Australia’s vast network of participants is likely to have increased the reach to potential participants, it may have also biassed the perspectives reported. In particular, interviewees interested in research participation may be more likely to seek out information themselves and therefore have a better experience of meeting their information needs.

### Implications for future research and daily practice

Future studies should examine how to best structure information delivery for rare cancer survivors, ensuring specific information needs and patient questions are addressed. Survivors should also be provided with necessary psychological support when information-seeking, where healthcare professionals may be best placed to refer their rare cancer patients to psychological evaluation and support. Future studies could then develop interventions that evaluate the psychological impact of limited information on rare cancers to ultimately help improve patient well-being and support systems.

## Conclusion

This study highlights the importance of rare cancer survivors having access to, and an understanding of, tailored, accurate information about their disease. Health professionals appreciate the complexity and uncertainty that comes with limited information, and understanding their patients’ experience of information-seeking may help providers to proactively question their patients’ unmet information needs and thereby respond with relevant, timely and desired information.

## Data Availability

No datasets were generated or analysed during the current study.

## Appendix 1. Interview guide for rare cancer survivors

- Welcome and introductions
- Purpose of the interview and how data will be used
- Guidance for interviewee, including emphasising the voluntary nature of the interview
- Topics to discuss, including:
  - ⚬ Changing needs throughout the cancer trajectory
  - ⚬ Satisfaction with information provided throughout cancer diagnosis
  - ⚬ Preferences for information delivery
  - ⚬ Development of an intervention that improves unmet information needs
- Closing the interview 

## Appendix 2. Interview guide for healthcare professionals

- Welcome and introductions
- Purpose of the interview and how data will be used
- Guidance for interviewee, including emphasising the voluntary nature of the interview
- Topics to discuss, including:
  - ⚬ Experience and expertise in rare cancer care
  - ⚬ Perspectives on information needs of rare cancer patients
  - ⚬ Preferences for information provision to rare cancer patients
  - ⚬ Perspectives on information-seeking behaviours of rare cancer patients
  - ⚬ Challenges of providing rare cancer information and potential strategies to overcome these
  - ⚬ Input to the development of an intervention that improves unmet information needs
- Closing the interview

## References

[1] Australian Institute of Health and Welfare. Cancer data in Australia 2024. Canberra: AIHW; 2024.

[2] de Heus E, Duijts SFA, van der Zwan JM, Kapiteijn E, Nieveen van Dijkum EJM, van Herpen CML, et al. The gap between rare and common cancers still exists: Results from a population-based study in the Netherlands. Eur J Cancer. 2022;167:103–11. 10.1016/j.ejca.2022.03.001 35421702

[3] Driehuis E, de Heus E, Schrieks M, Engelen V, Buffart TE, Vink GR, et al. Quality of life of patients with rare cancer: a comparison with patients with colorectal cancer and the association with disease trajectory-related factors. J Cancer Surviv. 2023;17(4):986–96. 10.1007/s11764-022-01324-7 36585575

[4] Blodt S, Kaiser M, Adam Y, Adami S, Schultze M, Muller-Nordhorn J, et al. Understanding the role of health information in patients’ experiences: secondary analysis of qualitative narrative interviews with people diagnosed with cancer in Germany. BMJ Open. 2018;8(3):e019576. 10.1136/bmjopen-2017-019576 PMC5857676 29530909

[5] ElKefi S, Asan O. How technology impacts communication between cancer patients and their health care providers: a systematic literature review. Int J Med Inform. 2021;149:104430. 10.1016/j.ijmedinf.2021.104430 PMC8131252 33684711

[6] Pomey MP, Denis JL, Dumez V. Patient engagement: how patient-provider partnerships transform healthcare organizations*.* Palgrave Macmillan; 2019.

[7] Husson O, Mols F, Oranje WA, Haak HR, Nieuwlaat WA, Netea-Maier RT, et al. Unmet information needs and impact of cancer in (long-term) thyroid cancer survivors: results of the PROFILES registry. Psychooncology. 2014;23(8):946–52. 10.1002/pon.3514 24619907

[8] Beghean R, Coffey L. “You either sink or you swim, and you’re better off swimming”: A qualitative study exploring the self-management experiences of soft tissue sarcoma survivors. Eur J Oncol Nurs. 2021;55: 102062. 10.1016/j.ejon.2021.102062 34775165

[9] Leyden S, Kolarova T, Bouvier C, Caplin M, Conroy S, Davies P, et al. Unmet needs in the international neuroendocrine tumor (NET) community: assessment of major gaps from the perspective of patients, patient advocates and NET health care professionals. Int J Cancer. 2020;146(5):1316–23. 10.1002/ijc.32678 PMC7004101 31509608

[10] de Heus E, van der Zwan JM, Husson O, Frissen AR, van Herpen CML, Merkx MAW, et al. Unmet supportive care needs of patients with rare cancer: a systematic review. Eur J Cancer Care (Engl). 2021;30(6):e13502. 10.1111/ecc.13502 34409667

[11] Yatim F, Cristofalo P, Minvielle E. What are the unmet information needs of cancer patients? A qualitative study. Glob J Health Sci. 2017. 10.5539/gjhs.v9n12p114.

[12] Hayatt A, Shelly A, Cox R, Humphries E, et al. How can we improve information for people affected by cancer? A national survey exploring gaps in current information provision, and challenges with accessing cancer information online. Patient Educ Couns. 2022;105(8):2763–2770. 10.1016/j.pec.2022.04.009 35465976

[13] Jo HS, Park K, Jung SM. A scoping review of consumer needs for cancer information. Patient Educ Couns. 2019;102(7):1237–50. 10.1016/j.pec.2019.02.004 30772114

[14] Chua GP, Tan HK, Gandhi M. What information do cancer patients want and how well are their needs being met? Ecancermedicalscience. 2018;12: 873. 10.3332/ecancer.2018.873 PMC6214674 30483353

[15] van Vliet LM, Meijers MC, van Dulmen S, van der Wall E, Plum N, Stouthard J, et al. Addressing challenges in information-provision: a qualitative study among oncologists and women with advanced breast cancer. BMC Palliat Care. 2021;20(1):142. 10.1186/s12904-021-00836-w PMC8442372 34521393

[16] Vandeborne L, van Overbeeke E, Dooms M, De Beleyr B, Huys I. Information needs of physicians regarding the diagnosis of rare diseases: a questionnaire-based study in Belgium. Orphanet J Rare Dis. 2019;14(1):99. 10.1186/s13023-019-1075-8 PMC6500578 31054581

[17] Ashtari S, Taylor AD. The internet knows more than my physician: qualitative interview study of people with rare diseases and how they use online support groups. J Med Internet Res. 2022;24(8): e39172. 10.2196/39172 PMC9459833 36006679

[18] Long JC, Best S, Nic Giolla Easpaig B, Hatem S, Fehlberg Z, Christodoulou J, et al. Needs of people with rare diseases that can be supported by electronic resources: a scoping review. BMJ Open. 2022;12(9):e060394. 10.1136/bmjopen-2021-060394 PMC9438091 36581982

[19] Ng CA, Luckett T, Mulhern B, Kee D, Lai-Kwon J, Joshua AM. What matters most to people with metastatic uveal melanoma? A qualitative study to inform future measurement of health-related quality of life. Melanoma Res. 2024;34(3):248–57. 10.1097/CMR.0000000000000961 PMC11045555 38469755

[20] Budych K, Helms TM, Schultz C. How do patients with rare diseases experience the medical encounter? Exploring role behavior and its impact on patient-physician interaction. Health Policy. 2012;105(2–3):154–64. 10.1016/j.healthpol.2012.02.018 22464590

[21] Stanze H, Schneider N, Nauck F, Marx G. “I can’t get it into my head that I have cancer...”-a qualitative interview study on needs of patients with lung cancer. PLoS ONE. 2019;14(5): e0216778. 10.1371/journal.pone.0216778 PMC6516640 31086395

[22] Lange-Drenth L, Schulz H, Endsin G, Bleich C. Patients with cancer searching for cancer- or health-specific web-based information: performance test analysis. J Med Internet Res. 2021;23(8):e23367. 10.2196/23367 PMC8406111 34398801

[23] Ziegler E, Hill J, Lieske B, Klein J, dem OVK, Kofahl C. Empowerment in cancer patients: does peer support make a difference? A systematic review. Psychooncology. 2022;31(5):683–704. 10.1002/pon.5869 34981594

[24] Boyes A, Turon H, Hall A, Watson R, Proietto A, Sanson-Fisher R. Preferences for models of peer support in the digital era: a cross-sectional survey of people with cancer. Psychooncology. 2018;27(9):2148–54. 10.1002/pon.4781 29808504

[25] Westendorp J, Geerse OP, van der Lee ML, Schoones JW, van Vliet MHM, Wit T, et al. Harmful communication behaviors in cancer care: a systematic review of patients and family caregivers perspectives. Psychooncology. 2023;32(12):1827–38. 10.1002/pon.6247 37957777

[26] Farrugia T, Duijts SFA, Wilson C, Hemming L, Cockburn C, Spelten E. Addressing cancer survivors’ information needs and satisfaction: a systematic review of potential intervention components for survivors with a rare cancer type. Orphanet J Rare Dis. 2024;19(1):387. 10.1186/s13023-024-03403-7 PMC11488126 39425097

[27] Chua GP, Tan HK, Gandhi M. Information sources and online information seeking behaviours of cancer patients in Singapore. Ecancermedicalscience. 2018;12: 880. 10.3332/ecancer.2018.880 PMC6214676 30483360

[28] Li J, Luo X, Cao Q, Lin Y, Xu Y, Li Q. Communication needs of cancer patients and/or caregivers: A critical literature review. J Oncol. 2020;2020: 2020:7432849. 10.1155/2020/7432849 PMC7229568 32454826

[29] Simons BCR, Hillen MA, Aarts JWM, Tromp JM, de Heus E, Duijts SFA. Disentangling trust of patients with rare cancer in their healthcare professionals and the healthcare system: a qualitative interview study. J Cancer Surviv. 2024. 10.1007/s11764-023-01531-w. PMC12081587 38225524

[30] Drabbe C, Grunhagen DJ, Van Houdt WJ, Braam PM, Soomers V, Van der Hage JA, et al. Diagnosed with a rare cancer: Experiences of adult sarcoma survivors with the healthcare system-results from the SURVSARC study. Cancers (Basel). 2021;13(4):679. 10.3390/cancers13040679 PMC7914609 33567553

